# Polymer Gel with Tunable Conductive Properties: A Material for Thermal Energy Harvesting

**DOI:** 10.1021/acsomega.2c05301

**Published:** 2022-12-13

**Authors:** Evgenia Vaganova, Dror Eliaz, Gregory Leitus, Aleksei Solomonov, Faina Dubnikova, Yishay Feldman, Irit Rosenhek-Goldian, Sidney R. Cohen, Ulyana Shimanovich

**Affiliations:** † Department of Molecular Chemistry and Materials Science, Weizmann Institute of Science, Rehovot7610001, Israel; ‡ Chemical Research Support Department, Weizmann Institute of Science, Rehovot7610001, Israel; § Department of Chemistry, The Hebrew University of Jerusalem, Jerusalem91904, Israel

## Abstract

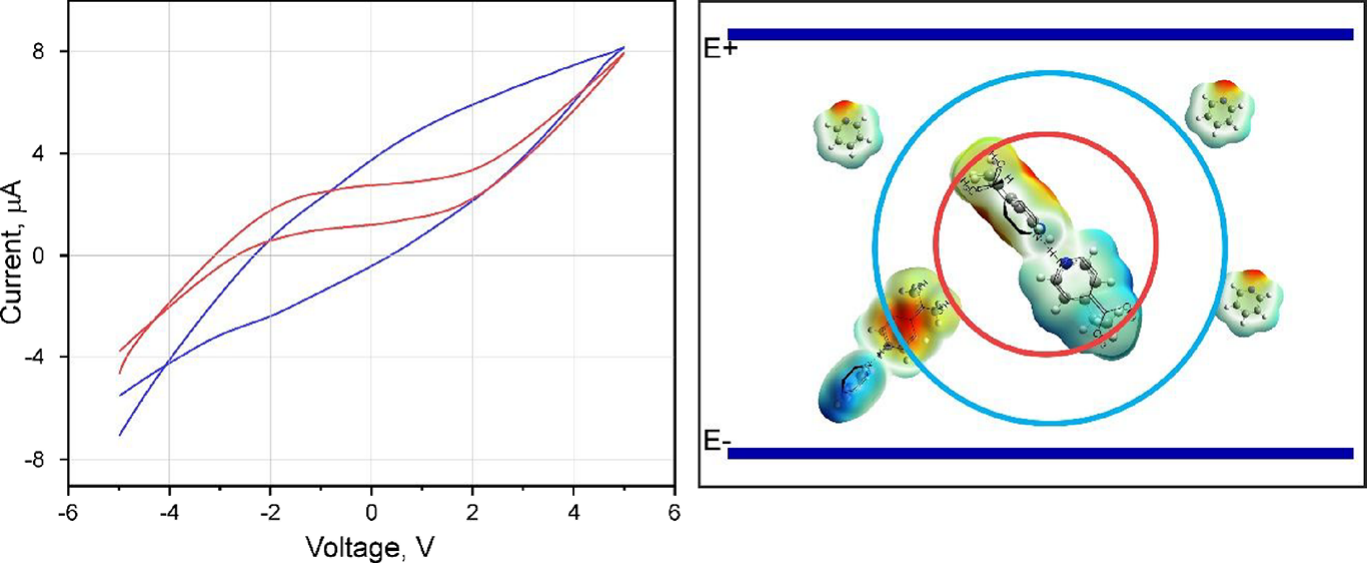

The spontaneous gelation of poly(4-vinyl pyridine)/pyridine solution produces materials with conductive properties that are suitable for various energy conversion technologies. The gel is a thermoelectric material with a conductivity of 2.2–5.0 × 10^–6^ S m^–1^ and dielectric constant ε = 11.3. On the molecular scale, the gel contains various types of hydrogen bonding, which are formed via self-protonation of the pyridine side chains. Our measurements and calculations revealed that the gelation process produces bias-dependent polymer complexes: *quasi-symmetric*, strongly *hydrogen-bonded* species, and weakly bound *protonated* structures. Under an applied DC bias, the gelled complexes differ in their capacitance/conductive characteristics. In this work, we exploited the bias-responsive characteristics of poly(4-vinyl pyridine) gelled complexes to develop a prototype of a thermal energy harvesting device. The measured device efficiency is *S =* Δ*V*/Δ*T* = 0.18 mV/K within the temperature range of 296–360 K. Investigation of the mechanism underlying the conversion of thermal energy into electric charge showed that the heat-controlled proton diffusion (the Soret effect) produces thermogalvanic redox reactions of hydrogen ions on the anode. The charge can be stored in an external capacitor for heat energy harvesting. These results advance our understanding of the molecular mechanisms underlying thermal energy conversion in the poly(4-vinyl pyridine)/pyridine gel. A device prototype, enabling thermal energy harvesting, successfully demonstrates a simple path toward the development of inexpensive, low-energy thermoelectric generators.

## 1 Introduction

Thermally generated electricity is currently a growing field with thermoelectric energy harvesting devices (THEDs) being able to store heat energy as thermally generated charge carriers, which can possibly be used for residential electricity needs. Heat from household appliances and even body heat are being considered for generating electricity.^1−5^ The heart of a THED is a thermoelectric generator (TEG), which converts thermal energy to an electric charge. Developing materials capable of such conversion is central to the field.^6−8^ Lightly doped semiconductors have been found to be the most efficient materials on which TEGs are based on. Bi_2_Te_3_ and its alloys display the highest known thermoelectric efficiency at room temperature. Mixed ionized and deformation potential scattering models,^9^ based on Bi_2_Te_3_, have been fitted to experimental thermoelectric data. The efficiency of Bi_2_Te_3_ was traced to high band degeneracy, low effective mass, high carrier mobility, and relatively low lattice thermal conductivity.^10^ However, these materials are not environmentally friendly. Furthermore, the alloying technology has a detrimental effect on charge mobility, and there is an urgent need for affordable, nontoxic materials for TEGs.^10^

Present stage polymers appropriate for TEGs are divided into three main groups: doped conductive polymers,^11−16^ polymer electrolytes,^17−22^ and conjugated polyelectrolytes.^23,24^ Examples include poly(diallyldimethylammonium chloride), an anionic polymeric electrolyte with a Seebeck coefficient *S* = 19.0 mV/K,^22^ and the copolymer poly(vinylidene fluoride-*co*-hexafluoropropylene) (matrix) with the ionic liquid 1-ethyl-3-methylimidazolium bis(trifluoro-methylsulfonyl)imide acting as the electrolyte.^19^ The Seebeck coefficient for this TEG can range from −4 to +14 mV/K.

Recently, a new class of thermoelectric polymers has been reported: ionic conductive polymers and ionogels proposed for low-grade heat harvesting.^25,26^

Here, we describe TEG/THED based on a stable, dopant-free, polymeric material, poly(4-vinyl pyridine) (P4VPy), gelled in liquid pyridine. The P4VPy gel has been shown to be a uniquely photosensitive material with highly reproducible electrical characteristics,^27−36^ while its structural^28,37^ and functional properties (light sensitivity and electrical conductivity) depend on the irradiation wavelength.^29,34,36−38^

The THED was designed as follows: a thin layer of the P4VPy gel is placed between indium-tin-oxide (ITO)/glass electrodes, a 10*V*_DC_ bias is supplied, and an external capacitor is connected in parallel. Upon application of low-level heat, a current is generated and the capacitor is charged, giving a Seebeck coefficient *S* of 0.18 mV/K over the temperature range of 298–363 K. To fully characterize and understand the gel properties underlying its THED operation, we used *I*–*V* electrical characterization, impedance measurements, atomic force microscopy (AFM) conductivity, transmission electron microscopy (TEM), X-ray diffraction (XRD), mass spectroscopy, and density functional theory (DFT) calculations.

## 2 Experimental Section

### 2.1 Gel Preparation

The P4VPy gel was prepared according to a standard procedure.^28^ P4VPy with an average molecular weight of 50,000 (Polysciences, Inc.) was dried in a vacuum oven (10^–3^ Torr) at room temperature for approximately 1 week prior to use. The pyridine (Py) solvent was anhydrous (<0.003% water, Aldrich). P4VPy was mixed with Py at a 1:1 ratio between the free solvent and the side-chain Py groups. For AFM and TEM imaging, the gel was diluted in anhydrous ethanol (Bell-Lab Ltd.).

### 2.2 THED

#### 2.2.1 Sample Preparation

The polymer gel was stored for approximately 1 week in the dark at room temperature prior to use. Fourier transform infrared (FTIR) spectroscopy showed no changes in the absorption spectra a week after preparation (data not shown). For electrical measurements, the gel was then spread on the conductive surface of a glass/ITO electrode (ITO-coated substrate, Merck) to a thickness of 0.25 mm. The bottom electrode was the anode, whereas a second glass/ITO electrode (the cathode) covered the gel. The distance between the electrodes and the size of the contact area was controlled by polyethylene terephthalate inserts to 0.5 cm × 0.5 cm × 0.25 mm.

#### 2.2.2 THED Operation

**Figure 1:**
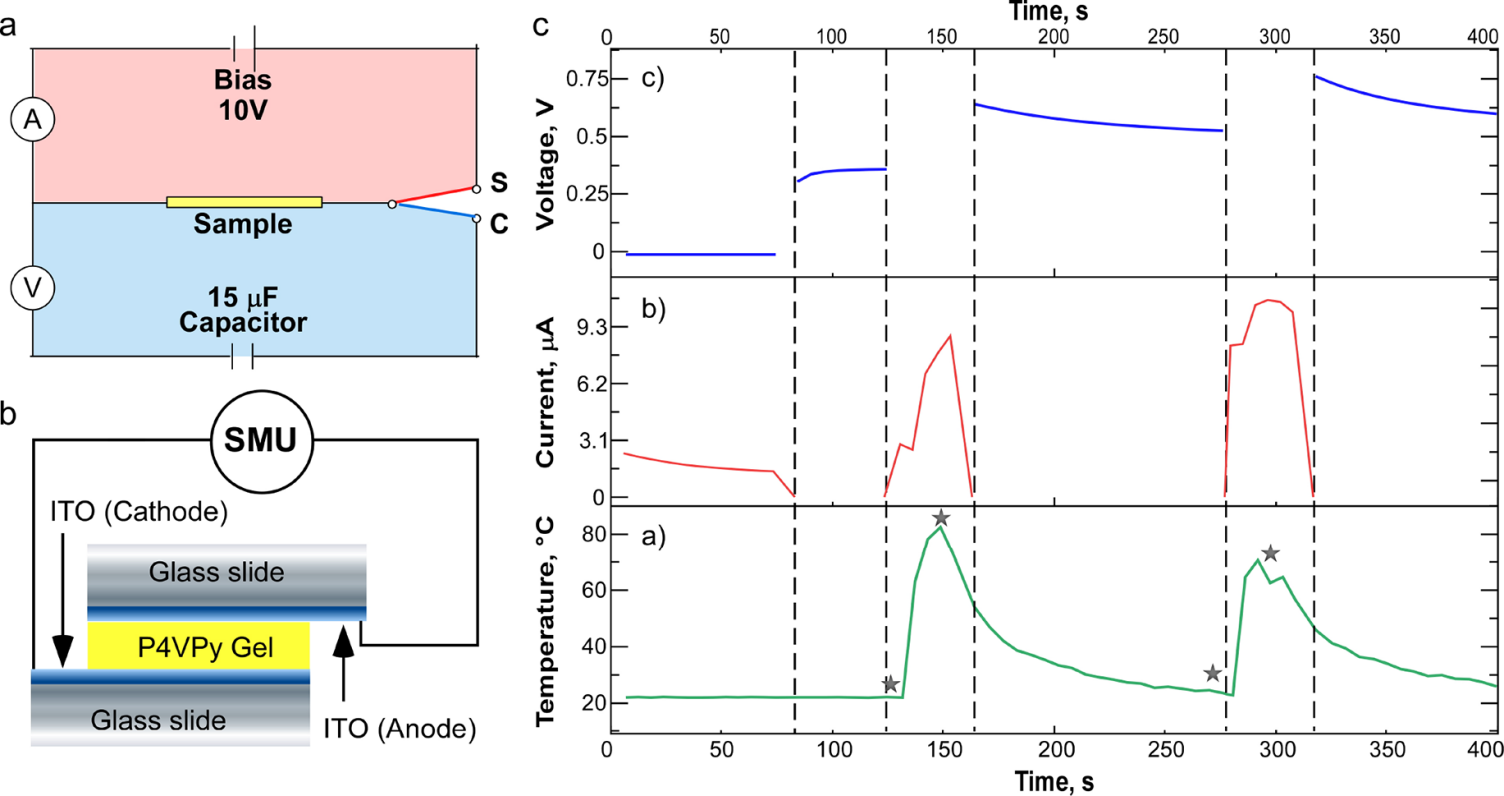
(a) Schematic representation of the design for a THED. Two sections of the device are connected by the sample (the yellow rectangle) and physically separated by a switch with two positions: **S** and **C**. In the **S** position (the pink section), a circuit is formed for current measurements through the sample; the **C** position (the blue section) forms a circuit for voltage measurements across the EC. (b) Scheme of the sample design: the P4VPy gel was placed between two glass slides, covered by conductive layers of ITO face to face to the gel. ITO electrodes are connected to the SMU. (c) The current was modulated by heat. (a) Change in voltage measured across the remote storage capacitor (EC) (15 μF) with time (switch in position C). (b) Change in electric current with time (switch in position S). (c) Change in the P4VPy gel sample temperature with time. The duration of heating was 10 s (on/off denoted by black stars), and the interval between applications of heating pulses was 100 s. The room temperature was 23 ± 0.1 °C. The accuracy of *T* measurement is equal to ±2.2%, of voltage ±0.12%, and of current ±0.05%. Therefore, the uncertainty of *S* (Seebeck/thermal coefficient) measurements is ≈ ±5%.

As mentioned, the THED (scheme presented in Figure 1a) consisted of two sections connected by the sample and electrically separated by a switch with two positions: **S** and **C**. When the switch was in the **S** (sample) position, the upper section including the sample and DC supply 10 V (or at 3.7 V as in Figure S1) formed a working circuit and the current through the sample was measured. A representative operation cycle is shown in Figure 1c.

For the first 80 s, the current through the sample was measured (10*V*_DC_). When the current stabilized, the sample was heated by a hot airflow (hair drier, 1750–2000 W) for 10 s. A standard thermocouple (IR2508BR) was placed on the upper electrode of the sample. During the 10 s of heating, the temperature of the sample reached 70–90 °C and the current increased. After 10 s, the DC voltage and heat flow were interrupted. The switch was turned to position **C** to measure the voltage across the external capacitor (EC). The sample and EC comprised a second circuit. The potential difference across the gel led to a current that charged the EC. The voltage across the capacitor was measured.

Next, for 100 s, the voltage on the EC was stabilized, and the temperature of the sample decreased and stabilized at 23 ± 0.5 °C (room temperature).

In the Supporting Information, the results of experiments with 20 s heating and 50 s interval between heating are presented (Figure S1).

This procedure was repeated for different cycling and heating times. *I*–*V* measurements were performed using a Janis ST-500-2 probe station in a two-electrode configuration.

#### 2.2.3 Electrical Characterization

*I*–*V* measurements were performed, as described above. Copper wires were connected to the ITO conductive surfaces. DC and AC voltages were provided by a Keithley 4200A-SCS system controlled by Clarius software. Our SCS system includes four 4200-SMUs (source measure units) that connect to the sample via 4200-PA Remote Preamplifiers, as well as a 4210-CVU (capacitance–voltage unit). The designations of all devices mentioned above are standard. DC *I*/*V* was measured with a 4200-SMU between −5 and +5 V. AC impedance was measured using a 4210-CVU (capacitance/voltage unit) with a 100 mV RMS amplitude and a frequency ranging from 1 kHz to 10 MHz. Different sweep rates were applied. To confirm the repeatability of the results, 5–10 replicate measurements were carried out.

Complex relative permittivity dependence on AC frequencies shows the difference in frequency behaviors for ε′_r_ (the real part) and ε″_r_ (the imaginary part). , where *A* is the area of one plate and *d* is the distance between the plates, and *C*, the measured capacitance, depends on the ion diffusion processes. ε″_r_ = σ/ωε_0_ is the lossy permittivity, where σ is the conductivity and ω = 2π*f* is the angular frequency. Therefore, the imaginary part (the loss factor) is related to conductivity.

### 2.3 AFM

#### 2.3.1 AFM Imaging

Approximately 2 mg of gel was dissolved in 2 mL of anhydrous ethanol. A drop of this solution was placed on a mica sheet and dried. Next, the sample was imaged by AFM (JPK Nanowizard 4 AFM, Germany) using AC240 cantilevers (Olympus, Tokyo, Japan; nominal resonance frequency of 70 kHz and spring constant of 2 N/m) in AC mode. The resolution of scan for 5 μm × 5 μm was 512 × 512 pixels. The images were processed using Gwyddion (64 bit) software^39^ for data visualization and ImageJ software for statistical analysis.

#### 2.3.2 AFM Current Measurements

For microsphere sample preparation, 1–2 mg of gel was dissolved in 2 mL of anhydrous ethanol and centrifuged in Minispin Plus (Eppendorf) at 5000 rot/s and 20 μL of the supernatant diluted in 1 mL of ethanol. A drop of the solution was placed on a gold-coated (50 nm) Si/SiO_2_ substrate (orientation 100, resistivity >1 Ohm·m). AFM current measurements on gel microspheres were performed using a MultiMode AFM with Nanoscope V electronics (Bruker AXS SAS, Santa Barbara, CA, USA). Scans were made using the PeakForce TUNA module with a HA_NC/W_2_C+ probe (ScanSens GmbH, Bremen, Germany) with a nominal spring constant of 12 N/m. Image processing was performed using Gwyddion (64 bit) software^41^ and Origin 2018.

### 2.4 XRD

XRD of both non-irradiated and irradiated materials was carried out in reflection geometry using a TTRAX III (Rigaku, Japan) theta–theta diffractometer equipped with a rotating Cu anode operating at 50 kV and 200 mA. A bent graphite monochromator and a scintillation detector were aligned in the diffracted beam, and θ/2θ scans were performed under specular conditions in the Bragg–Brentano mode with variable slits. The 2θ scanning range was 1–50 degrees with a step size of 0.025 degrees and a scan speed of 0.4 degree per minute.

### 2.5 TEM

TEM measurements were done using an FEI Tecnai G2 F20 TEM microscope, operated at 200 kV. Grids were prepared using the drop-cast method (the solution was sonicated, and then a droplet was placed on the grid and dried in an ambient atmosphere).

### 2.6 Mass Spectrometry

A Waters SQ Detector 2 (Manchester, UK) with an electrospray ionization (ESI) interface in a positive ionization mode provided the mass spectra from the *m*/*z* range of 40 to 2100. The parameters were set as follows: capillary voltage at 2.8 kV, cone gas flow at 50 L/h, source temperature at 120 °C, and cone voltage at 20 and 40 V. The desolvation temperature was set at 200 °C, and the desolvation gas (N_2_) flow rate was set at 350 L/h. The sample solution was introduced by direct syringe infusion at a flow rate of 10 μL/min. The scan duration was 2.5 s, and eight scans were combined to produce a spectrum. Waters MassLynx v4.2 software was used for data acquisition and processing.

The samples were injected for ionization in the following order: MeOH as a blank, followed by a sample. The spectra were obtained with the same ionization energy (cone voltage). Samples and blanks were analyzed in both positive and negative ionization modes. Since there were no peaks in the negative mode that differed from the blank spectrum, the results are summarized only for the positive ionization mode.

## 3 Results and Discussion

### 3.1 Thermal Energy Harvesting by Conversion of Heat into an Electric Charge

The principal electrical scheme of the THED is shown in Figure 1a–c. The THED working principle is described in the Experimental Section. In short, here, we have utilized a two-component polymer gel (P4VPy) for the design and construction of the THED capable of efficiently collecting and converting thermal energy into an electric charge. As described in the Experimental Section, the THED (Figure 1a) is composed of two operation parts, forming two distinct operation circuits: TEG and EC (external capacitor for charge accumulation). The TEG sample comprised the P4VPy gel placed between two ITO glass slides.

The initial conditions for THED operation were room temperature 23 ± 0.5 °C, and the current measured through the sample was 2.5 μA (10*V*_DC_). After heating for 10 s, the sample temperature increased to 91 °C and the current increased to 8.5 μA (Figure 1a,b). The switch was then turned to position **C** and the voltage across the EC was recorded as the sample cooled for 100 s. A saturated capacitor voltage of 0.57 V was measured. The procedure was then repeated. For the next 10 s heating cycle, the sample temperature rose from 23 to 71 °C, and the current through the sample reached 10.9 μA. Correspondingly, the voltage through the EC increased and stabilized at 0.62 V. The capacitor charged to a potential of 1 V when the heating was 20 s and the interval between heating was 50 s (Figure S1).

The capacitance of the sample *C* = 0.1 nF was evaluated. Overall, our results indicate that (1) the temperature controls an electric current through the P4VPy gel and (2) TEG discharging led to charging of the EC, accompanied by a voltage rise.

Evaluation of the THED efficiency (*S*) as represented by the Seebeck coefficient is determined from eq 1:
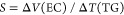
1

Here, Δ*V*(EC) is the voltage on EC following TH (sample) discharging. As we are unable to measure this value directly across the sample, it is measured on the capacitor, for which the asymptotic voltage must equal that across the sample. Δ*T*(TG) is the temperature difference on the sample due to heating. *S* ranges between 70 and 180 μV/K under heating within a temperature range of 296–360 K.

Thus, the gel is thermoelectric and suitable for the transfer of low energy heat to stored charge on a level of a modern TEG.^22^

The physical characteristics of the material (dielectric constant ε = 11.3 (Experimental Section), electrical conductivity 2.2–5.0 × 10^–6^ S m^–1^ (Figure S2), and activation energy of thermal electrical conductivity, calculated by the Arrhenius equation, equal to 0.7 eV^40^ (Figure S3)) describe the dielectric properties of the gel on a level of typical semiconducting materials.

To further understand the mechanism of gel thermoelectricity, its structural and electrical properties have been studied. Additionally, DFT was applied to model the molecular-level events.

### 3.2 XRD, TEM, and AFM

**Figure 2:**
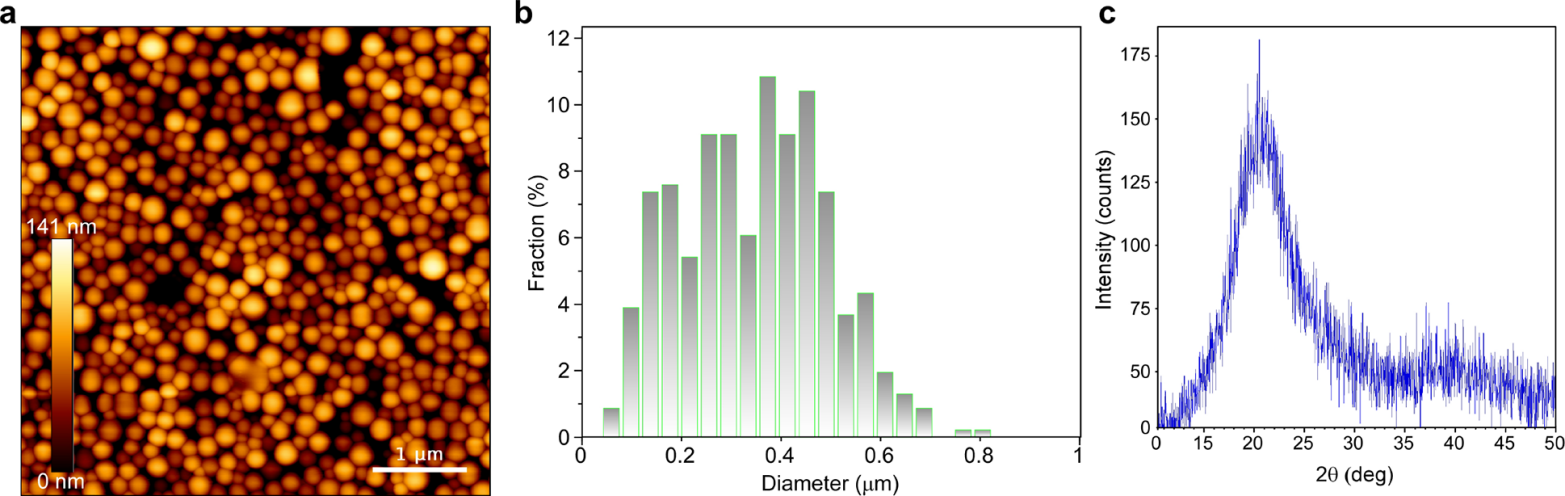
(a) AFM image of a P4VPy gel. The scale bar is 1 μm. (b) Analysis of microsphere diameter distribution from TEM images (TEM microsphere images are shown in Figure S4; height and corresponding phase images are shown in Figure S5). (c) XRD diffraction pattern of the P4VPy gel.

The morphology of the gel was determined by AFM and TEM (preparation described in the Experimental Section). The analysis revealed that the gelled structure formed into microspheres (Figure 2a,b). AFM height and phase analyses are shown in Figure S5.

The measured diameter of the microspheres ranged between 0.1 and 0.8 μm, as shown in Figure 2b. Further structural analysis of the gelled sample, using XRD (Figure 2c), showed that the gel structure was amorphous, with an average distance between organic molecules of 4 angstroms.

### 3.3 AFM Conductivity Measurements

**Figure 3:**
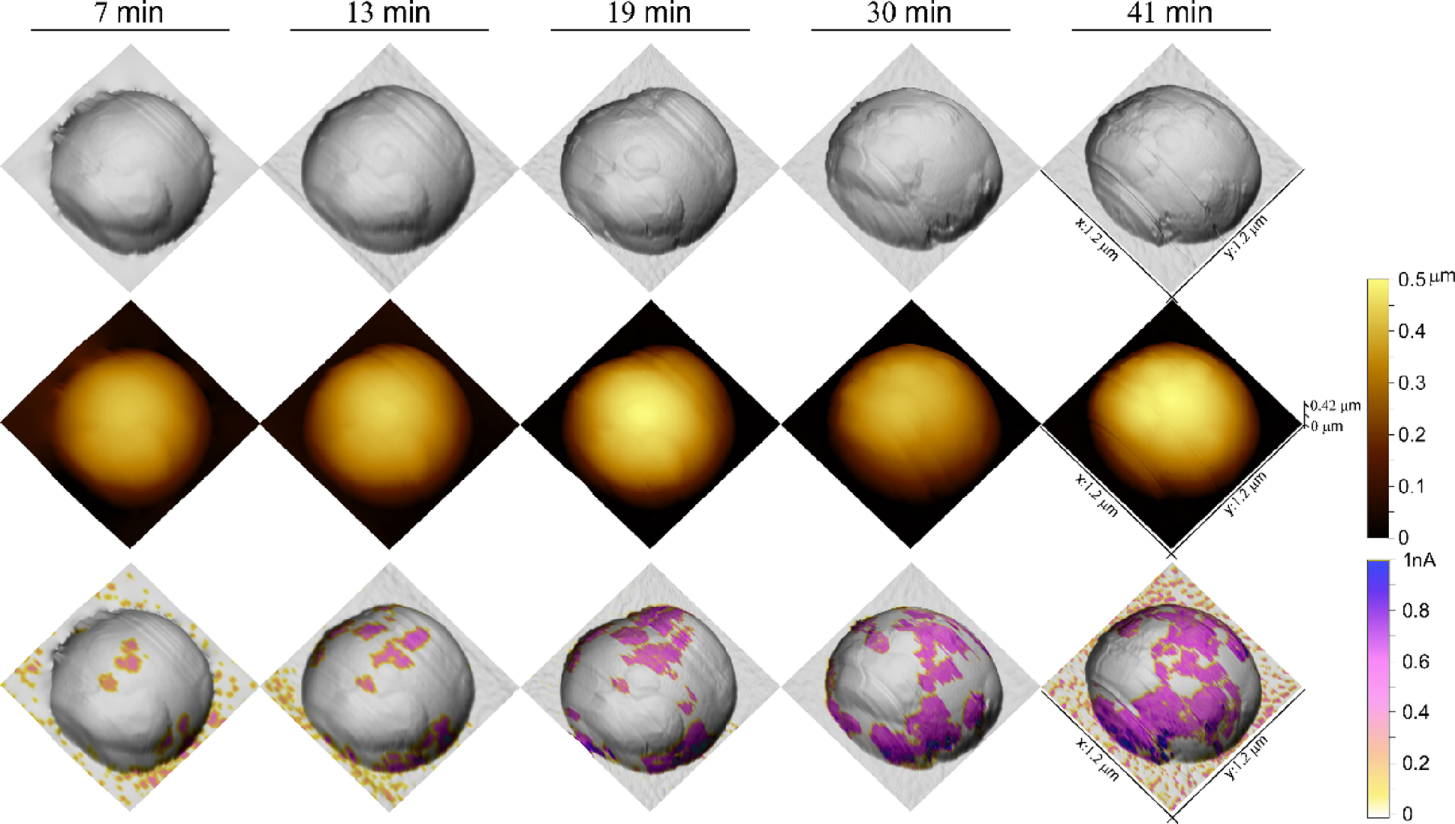
AFM images showing repeated scans on a single polymeric microsphere measured under constant 3*V*_DC_ bias at the indicated times. First row: AFM error signal accentuates the local morphology of the microspheres. Second row: 3D height images show the vertical scale. Third row: current maps are overlaid on the error signal image. The expansion of the conducting region can be explained by polarization as a result of the applied electric field. Corresponding images at each time were collected simultaneously from different data channels.

Application of a constant voltage (3*V*_DC_) through the microspheres caused the gradual appearance of a current through the microsphere structure. Typical morphology and conductivity maps measured on a representative microsphere are depicted in Figure 3. The current mapping indicated the presence of conducting patches, which are dynamic in their nature and tend to grow and merge with continued scans. Overall, the microspheres are conductive with a measured average current, over the conductive patches, of 0.4 ± 0.1 nA.

The growth of conductive patches on the surface is ascribed to inherent properties of dielectrics—electrical polarization.

### 3.4 P4VPy Electrical Characteristics

**Figure 4:**
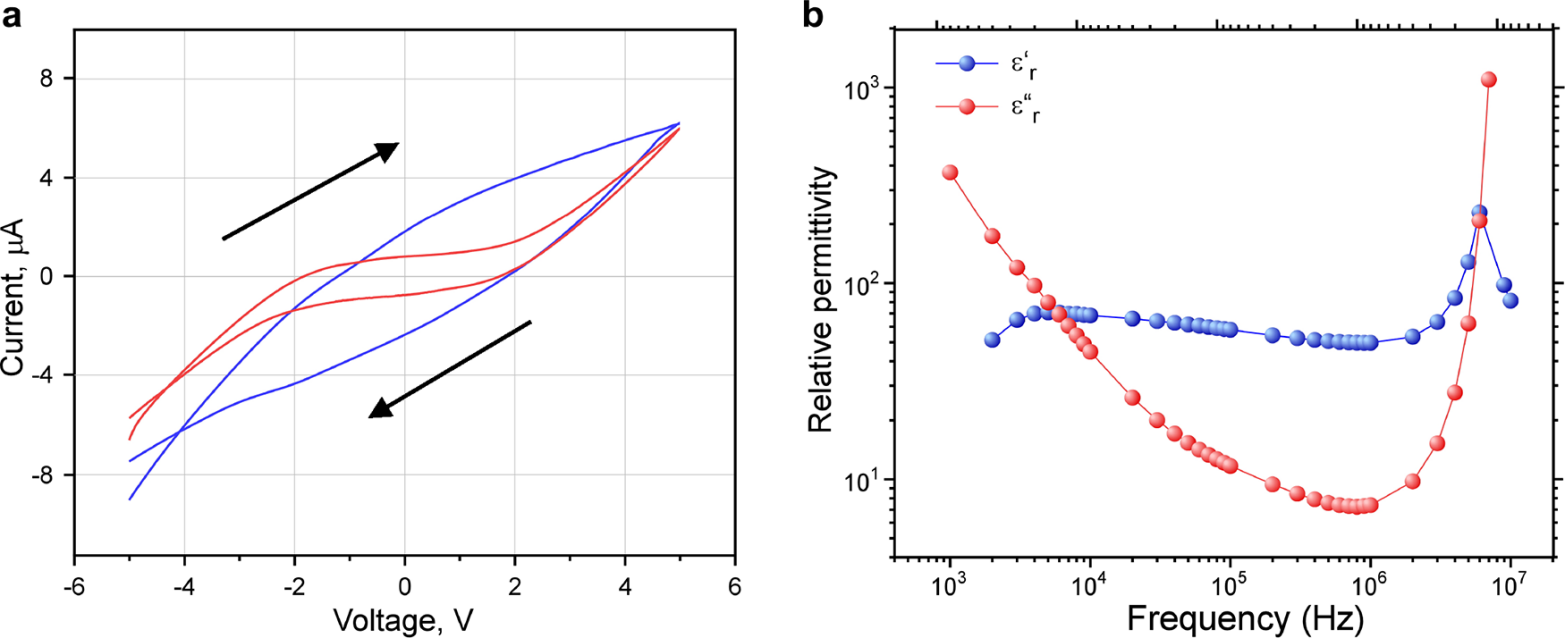
(a) Room temperature cyclic voltammetry measurements (*I*–*V*) for a typical poly(4-vinyl pyridine)/pyridine gel sample with a sweep rate of 1.5 V/s (blue curve) and 0.20 V/s (red curve). (b) Complex relative permittivity dependence on AC frequencies; ε′_r_ is the real part (blue) and ε″_r_ is the imaginary part (red) of the relative permittivity. Measurements were conducted at room temperature; the AC frequency range was 10 kHz to 10 MHz, and AC voltage RMS *V*_AC_ = 100 mV.

To elucidate the mechanism underlying P4VPy electrical conductivity, cyclic voltammetry and impedance measurements were conducted (Figure 4a,b). We observed that the cyclic voltammetry behavior of the gel is strongly dependent on the voltage sweep rate. The gel cyclic voltammetry measurements were carried out in a voltage range of ±5 V. The voltage sweep rate was varied from 0.05 to 1.60 V/s (Figure S6). Typical cyclic voltammograms (CV) are shown in Figure 4a as the blue plot (faster voltage sweep rate of 1.6 V/s) and red plot (0.20 V/s). The polarization effect manifests itself in the deviation from linear *I*–*V* dependence. For the fast scan, the current was 0 at a voltage of −1.17 V for negative to positive scans and at +2.0 V for positive to negative scans. Surprisingly, for the slow scan regime, two characteristic regions appear: capacitive mode in the range ±2 V and conductive mode showing a quasi-linear dependence outside of this range.

The evaluated conductivity, namely, the average resistance over the full range of the current vs voltage plot (sweep rate = 0.05–1.6 V/s), was observed to be within the range of 2.2–5.0 × 10^–6^ S m^–1^ (Figure S6). All slow and fast voltammograms are reversible and reproducible.

A study of the ionic conductivity in the gel was done previously by photoinduced pH changes.^29^ Under direct irradiation at the proton transfer center (385 nm wavelength), a reversible, photoinduced drop in the pH from 9.1 to 8.4 in ref (29) clearly identified the ionic species as protons.

Nonlinear *I*–*V* dependence (Figure 4a and Figures S2 and S6) complements each other and shows the gel to be a complex material, combining properties of spontaneously formed proton polyelectrolyte and polarizable capability.

The electrical properties of the gel as a function of frequency (impedance spectroscopy) were studied to differentiate between the electric field effect and the ionic and polar features of the material.^41−44^ We measured and analyzed the permittivity of the P4VPy gel (Figure 4b). The real part is responsible for the ionic processes, and it weakly depends on the AC electric field frequency. Some deviation from linearity at about 1 kHz and 6 MHz can be observed. The imaginary part gradually decayed as the frequency rose until reaching a minimum at a frequency of 1 MHz, after which it increased rapidly so that at 10 MHz the value was greater than at 1 kHz.

From the results of the electrical measurements, we can conclude that (1) the gel is dielectric; (2) at high voltage sweep rates, the gel has ionic and polarizable properties; (3) at slow voltage sweep rates, distinctive capacitive and conductive modes appear with mode transition at ±2 V; (4) AC measurements confirm that capacitive (independent of frequency) and conductive (frequency dependent) modes exist in the gel; (5) the gel is amorphous on the molecular level, and its macrostructure is microspheres; (6) the gel microspheres are conductive; and (7) the area of conductive patches grows with continued DC field application.

Structural analysis of the gel using FTIR spectroscopy revealed that on a molecular level, the gel contains various types of hydrogen bonding, which are formed via self-protonation of the pyridine side chains. The self-protonation of the pyridine side chains was experimentally proven by DFT modeling explained earlier.^33^ The two FTIR absorption bands at 3400 cm^–1^ (strength of hydrogen bonding *E* ∼4–8 kJ/mol) and a low-intensity, broad absorption centered at 1700 cm^–1^ (*E* ∼50 kJ/mol) accompanying the gelation were discussed in several publications.^27,30−32^ The FTIR band at 3400 cm^–1^ was assigned to the weakly bonded *protonated* complex of a self-protonated pyridine side chain with pyridine. The low-intensity FTIR band, at 1700 cm^–1^, was assigned to the tail-to-tail (N•••H•••N), strongly *quasi-symmetric* hydrogen-bonded self-protonated pyridine side chain with a side-chain complex (Supporting Information, Figure S7).

Further structural analysis by mass spectrometry (Figure S8) confirmed the presence of dimers in the gel. The peak at 160 *m*/*z* was assigned to a hydrogen-bonded pyridine-to-pyridine (Py•••H•••Py) dimer, with the pyridine dimer (Py)_2_ appearing at 159.19 *m*/*z*.

To evaluate the role of weakly and strongly hydrogen-bonded dimers in thermoelectric energy transfer properties, the physical properties of those dimers were investigated by DFT.

### 3.5 DFT Calculations

Modeling of molecular gel dimers was done with DFT. In DFT (SI), isopropylpyridine was used as a model for a P4VPy side chain. The DFT calculations confirmed that two molecular dimers, *protonated* (PP4-sp/py) and *quasi-symmetric* hydrogen bonded (PP4-sp/PP4) described earlier,^31,33^ were thermodynamically favorable (Table S1).

We postulate that these gel entities are the species responsible for the DC field-induced polarized elements. These two dimers (PP4-sp/py and PP4-sp/PP4) should differ in their polarization properties due to vastly different strengths of the hydrogen dimer bonds (4–8 and ∼50 kJ/mol, respectively). The different charge distributions of these gel active components including the dimers in the ground state are shown in the Supporting Information (DFT (SI); Figures S9 and S10).

Two different electrical modes are observed, capacitive and conductive, with a transition point at ±2 V (Figure 4a, red curve). The different modes appear in the permittivity measurements (Figure 4b) and could be explained by two different types of active polymer species in the gel.

The effect of DC field on the molecular polarity of PP4-sp/py was modeled by excited state dipole moment calculations and comparison with the dipole moment in the ground state (DFT (SII); Table S2). The excited triplet state T_0_ has a calculated dipole moment μ_T0_ = 20.3486 D, and the excited singlet state S_1_ has a dipole moment μ_S1_ = 20.3499 D. In comparison, the calculated dipole moment of the ground state S_0_ is μ_S0_ = 10.1295 D. Furthermore, the ionization potential was calculated as 5.7 eV, in comparison with 9.27 and 6.27 eV calculated for pyridine and a self-protonated pyridine side chain, respectively.^35^ The applied DC field (in experiments ±5 V at maximum) is sufficient to ionize PP4-sp/py.

The concentration of PP4-sp/py species in the gel can be estimated at 4–20% from Boltzmann distribution calculations (Supporting Information). The proton diffusion is enhanced by pyridine solvent molecules, which are known proton vehicles,^33^ and is temperature controlled.

The quasi-symmetric dimer PP4-sp/PP4 with its much larger hydrogen bond strength *E* ∼50 kJ/mol most probably cannot be aligned and cannot be ionized at the energy of the applied DC field. Nonetheless, the DC field can cause torque of the dimer with tail-to-tail pyridine rings to twist around the strong hydrogen bonds to align both rings in one plane. This can cause electron density redistribution to form a quinone-like conjugated structure with enhanced conductivity.^33^ The effect of oriented external electric fields on different polar/polarizable structures was described.^45^ Recently, it was shown that electric fields can act as a tweezer to control orientation or different reactions, depending on the ionic or covalent bond strength.^46^

The thermoelectric processes have been studied and can be described as follows: (1) weakly bound, protonated pyridine side-chain/pyridine complexes are ionized by an applied DC field; (2) a temperature increase enhances proton diffusion; (3) thermal conductivity of the material should be comparatively low due to the microspherical morphology; (4) diffused protons are deposited on the anode; (5) strongly bound quasi-symmetric side-chain polymer dimers form a conductive structure under an applied DC field; (6) thermogalvanic processes on the anode lead to capacitor discharge; and (7) discharging the TG capacitor charges the EC. This new TG is capable of forming a simple, effective THED with a Soret coefficient equivalent to that of modern polymeric materials.

## 4 Conclusions

Application of a DC field across a P4VPy gel induces polymer ionization and dipole polarization. Both properties stem from the presence of spontaneously organized dimers in the gel, including low-energy hydrogen-bonded self-protonated pyridine side chains with pyridine and quasi-symmetric hydrogen-bonded self-protonated pyridine side chains with pyridine side-chain dimers. The weakly hydrogen-bonded dimer undergoes ionization and liberates protons to enhance electronic conductivity on quasi-symmetric polymer side-chain dimers under an applied DC field. These modes were confirmed by cyclic voltammetry, impedance, and AFM conductivity measurements. Under the application of a nonhomogeneous thermal field, the ionic thermodiffusion process is responsible for the thermoelectric effect (the Soret effect). Electrochemical reduction controls the current on the anode, and polymer polarization controls the bulk conductivity. The gel’s conductivity is regulated by the applied temperature. Protonated side chains adopt a quinone-type structure and complete conjugation, enhancing the electron transfer.^33^ Applied heat leads to a flow of current, charging the EC due to the difference in the ionization potential between the TEG and the EC.

Thus, in this study, we demonstrated that the P4VPy gel, due to its exceptional thermoelectric characteristics, has been successfully implemented into a THED prototype for efficient conversion of thermal energy into an electric charge.

## Supporting Information Available

Time dependence of the voltage across the external capacitor, conductivity changes of the P4VPy gel under different temperatures, Arrhenius plot of the gel’s conductivity, TEM images of the polymeric microspheres, AFM images of the P4VPy gel, changes in conductivity of the P4VPy gel under different voltage sweep rates, FTIR spectrum of the P4VPy gel, mass spectroscopy spectra of 2–4 mg of a gel sample dissolved in 0.5 mL of MeOH in the dark, charge distribution for free molecules and in two dimers, illustrative sketch of the DC field-induced processes in microspheres, and tables for the summary of the results of quantum mechanical calculations and the calculated energy of the states (eV) and dipole moments (D) (PDF)

## References

[ref1] Teixeira J. S.; Costa R. S.; Pires A. L.; Pereira A. M.; Pereira C. Hybrid Dual-Function Thermal Energy Harvesting and Storage Technologies: Towards Self-Chargeable Flexible/Wearable Devices. Dalton Trans. 2021, 50, 9983–10013. 10.1039/D1DT01568K. 34264261

[ref2] Ando Junior O. H.; Maran A. L. O.; Henao N. C. A Review of the Development and Applications of Thermoelectric Microgenerators for Energy Harvesting. Renewable Sustainable Energy Rev. 2018, 91, 376–393. 10.1016/J.RSER.2018.03.052.

[ref3] Liu Y.; Wang H.; Sherrell P. C.; Liu L.; Wang Y.; Chen J. Potentially Wearable Thermo-Electrochemical Cells for Body Heat Harvesting: From Mechanism, Materials, Strategies to Applications. Adv. Sci. 2021, 8, 2100669 10.1002/ADVS.202100669.

[ref4] Kishore R.; Priya S. A Review on Low-Grade Thermal Energy Harvesting: Materials, Methods and Devices. Materials (Basel). 2018, 11, 1433 10.3390/ma11081433. 30110947 PMC6119907

[ref5] Nozariasbmarz A.; Suarez F.; Dycus J. H.; Cabral M. J.; LeBeau J. M.; Öztürk M. C.; Vashaee D. Thermoelectric Generators for Wearable Body Heat Harvesting: Material and Device Concurrent Optimization. Nano Energy 2020, 67, 104265 10.1016/J.NANOEN.2019.104265.

[ref6] Lee H. Thermoelectrics: Design and Materials; Lee H., Ed.; John Wiley & Sons, Ltd: Chichester, UK, 2016. 10.1002/9781118848944.

[ref7] Wang X.; Huang Y.-T.; Liu C.; Mu K.; Li K. H.; Wang S.; Yang Y.; Wang L.; Su C.-H.; Feng S.-P. Direct Thermal Charging Cell for Converting Low-Grade Heat to Electricity. Nat. Commun. 2019, 10, 4151 10.1038/s41467-019-12144-2. 31515483 PMC6742635

[ref8] Champier D. Thermoelectric Generators: A Review of Applications. Energy Convers. Manage. 2017, 140, 167–181. 10.1016/j.enconman.2017.02.070.

[ref9] Khan F. S.; Allen P. B. Deformation Potentials and Electron-Phonon Scattering: Two New Theorems. Phys. Rev. B 1984, 29, 3341 10.1103/PhysRevB.29.3341.

[ref10] Witting I. T.; Chasapis T. C.; Ricci F.; Peters M.; Heinz N. A.; Hautier G.; Snyder G. J. The Thermoelectric Properties of Bismuth Telluride. Adv. Electron. Mater. 2019, 5, 1800904 10.1002/aelm.201800904.

[ref11] Shirakawa H.; Louis E. J.; MacDiarmid A. G.; Chiang C. K.; Heeger A. J. Synthesis of Electrically Conducting Organic Polymers: Halogen Derivatives of Polyacetylene, (CH) X. J. Chem. Soc., Chem. Commun. 1977, 16, 578–580. 10.1039/c39770000578.

[ref12] Pron A.; Rannou P. Processible Conjugated Polymers: From Organic Semiconductors to Organic Metals and Superconductors. Prog. Polym. Sci. 2002, 27, 135–190. 10.1016/S0079-6700(01)00043-0.

[ref13] Toshima N. Conductive Polymers as a New Type of Thermoelectric Material. Macromol. Symp. 2002, 186, 81–86. 10.1002/1521-3900(200208)186:1<81::AID-MASY81>3.0.CO;2-S.

[ref14] Zhang Q.; Sun Y.; Xu W.; Zhu D. Organic Thermoelectric Materials: Emerging Green Energy Materials Converting Heat to Electricity Directly and Efficiently. Adv. Mater. 2014, 26, 6829–6851. 10.1002/ADMA.201305371. 24687930

[ref15] Yao C. J.; Zhang H. L.; Zhang Q. Recent Progress in Thermoelectric Materials Based on Conjugated Polymers. Polymer 2019, 11, 107 10.3390/POLYM11010107. PMC6401909 30960091

[ref16] Bubnova O.; Khan Z. U.; Malti A.; Braun S.; Fahlman M.; Berggren M.; Crispin X. Optimization of the Thermoelectric Figure of Merit in the Conducting Polymer Poly(3,4-Ethylenedioxythiophene). Nat. Mater. 2011, 10, 429–433. 10.1038/nmat3012. 21532583

[ref17] Guan X.; Yildirim E.; Fan Z.; Lu W.; Li B.; Zeng K.; Yang S.-W.; Ouyang J. Thermoelectric Polymer Films with a Significantly High Seebeck Coefficient and Thermoelectric Power Factor Obtained through Surface Energy Filtering. J. Mater. Chem. A 2020, 8, 13600–13609. 10.1039/D0TA05324D.

[ref18] Han C.-G.; Qian X.; Li Q.; Deng B.; Zhu Y.; Han Z.; Zhang W.; Wang W.; Feng S.-P.; Chen G.; Liu W. Giant Thermopower of Ionic Gelatin near Room Temperature. Science 2020, 368, 1091–1098. 10.1126/science.aaz5045. 32354840

[ref19] Zhao D.; Martinelli A.; Willfahrt A.; Fischer T.; Bernin D.; Khan Z. U.; Shahi M.; Brill J.; Jonsson M. P.; Fabiano S.; Crispin X. Polymer Gels with Tunable Ionic Seebeck Coefficient for Ultra-Sensitive Printed Thermopiles. Nat. Commun. 2019, 10, 1093 10.1038/s41467-019-08930-7. 30842422 PMC6403253

[ref20] Choi K.; Son J.; Park Y. T.; Cho J. S.; Cho C. Effect of the Conformation Changes of Polyelectrolytes on Organic Thermoelectric Performances. Macromol. Res. 2020, 28, 997–1002. 10.1007/s13233-020-8133-x.

[ref21] Jeong M.; Noh J.; Zahidul Islam M.; Kim K.; Sohn A.; Kim W.; Yu C.; Jeong M.; Noh J.; Yu C.; Islam M. Z.; Sohn A.; Kim K.; Kim W. Embedding Aligned Graphene Oxides in Polyelectrolytes to Facilitate Thermo-Diffusion of Protons for High Ionic Thermoelectric Figure-of-Merit. Adv. Funct. Mater. 2021, 31, 2011016 10.1002/ADFM.202011016.

[ref22] Kim S. L.; Hsu J.-H.; Yu C. Thermoelectric Effects in Solid-State Polyelectrolytes. Org. Electron. 2018, 54, 231–236. 10.1016/j.orgel.2017.12.021.

[ref23] Mai C.-K.; Schlitz R. A.; Su G. M.; Spitzer D.; Wang X.; Fronk S. L.; Cahill D. G.; Chabinyc M. L.; Bazan G. C. Side-Chain Effects on the Conductivity, Morphology, and Thermoelectric Properties of Self-Doped Narrow-Band-Gap Conjugated Polyelectrolytes. J. Am. Chem. Soc. 2014, 136, 13478–13481. 10.1021/ja504284r. 25179403

[ref24] Kee S.; Haque M. A.; Lee Y.; Nguyen T. L.; Rosas Villalva D.; Troughton J.; Emwas A.-H.; Alshareef H. N.; Woo H. Y.; Baran D. A Highly Conductive Conjugated Polyelectrolyte for Flexible Organic Thermoelectrics. ACS Appl. Energy Mater. 2020, 3, 8667–8675. 10.1021/acsaem.0c01213.

[ref25] Yang B.; Portale G. Ionic Thermoelectric Materials for Waste Heat Harvesting. Colloid Polym. Sci. 2021, 299, 465–479. 10.1007/S00396-020-04792-4.

[ref26] Zhao W.; Wang Z.; Hu R.; Luo X. Gel-Based Thermocells for Low-Grade Heat Harvesting. EPL 2021, 135, 26001 10.1209/0295-5075/AC2075.

[ref27] Vaganova E.; Rozenberg M.; Yitzchaik S. Multicolor Emission in Poly(4-Vinyl-Pyridine) Gel. Chem. Mater. 2000, 12, 261–263. 10.1021/cm990480x.

[ref28] Vaganova E.; Meshulam G.; Kotler Z.; Rozenberg M.; Yitzchaik S. Photoinduced Structural Changes in Poly(4-Vinyl Pyridine): A Luminescence Study. J. Fluoresc. 2000, 10, 81–88. 10.1023/A:1009426622243.

[ref29] Vaganova E.; Yitzchaik S. Tunable Emission in Poly(4-Vinylpyridine)-Based Gel. Acta Polym. 1998, 49, 637–641. 10.1002/(sici)1521-4044(199810)49:10/113.0.co;2-4.

[ref30] Vaganova E.; Yitzchaik S. Structural and Optical Properties of Poly(4-Vinyl Pyridine)/Pyridine Gels. Macromol. Symp. 2004, 207, 95–104. 10.1002/MASY.200450309.

[ref31] Rozenberg M.; Vaganova E.; Yitzchaik S. FTIR Study of Self-Protonation and Gel Formation in Pyridinic Solutions of Poly(4-Vinylpyridine). New J. Chem. 2000, 24, 109–111. 10.1039/a906937b.

[ref32] Vaganova E.; Rozenberg M.; Dubnikova F.; Danovich D.; Yitzchaik S. Acidity of the Methyne Group of Poly(4-Vinylpyridine) Leads to Side-Chain Protonation in Pyridine. New J. Chem. 2015, 39, 5920–5922. 10.1039/C5NJ01246E.

[ref33] Vaganova E.; Wachtel E.; Leitus G.; Danovich D.; Lesnichin S.; Shenderovich I. G.; Limbach H.-H.; Yitzchaik S. Photoinduced Proton Transfer in a Pyridine Based Polymer Gel. J. Phys. Chem. B 2010, 114, 10728–10733. 10.1021/jp104277r. 20666565

[ref34] Berestetsky N.; Vaganova E.; Wachtel E.; Leitus G.; Goldberg A.; Yitzchaik S. Photoactive Proton Conductor: Poly(4-Vinyl Pyridine) Gel. J. Phys. Chem. B 2008, 112, 3662–3667. 10.1021/jp711038u. 18318532

[ref35] Vaganova E.; Berestetsky N.; Yitzchaik S.; Goldberg A. Modelling of Poly(4-Vinyl Pyridine) and Poly(4-Vinyl Pyridine)/Pyridine Composites: Structural and Optical Properties. Mol. Simul. 2008, 34, 981–987. 10.1080/08927020802256041.

[ref36] Vaganova E.; Eliaz D.; Shimanovich U.; Leitus G.; Aqad E.; Lokshin V.; Khodorkovsky V. Light-Induced Reactions within Poly(4-Vinyl Pyridine)/Pyridine Gels: The 1,6-Polyazaacetylene Oligomers Formation. Molecules 2021, 26, 6925 10.3390/molecules26226925. 34834017 PMC8621047

[ref37] Vaganova E.; Meshulam G.; Kotler Z.; Yitzchaik S. Ion-Doping Role in Photoinduced Processes in Pyridine-Containing Polymeric Viscous Solutions. Polym. Adv. Technol. 2002, 13, 975–981. 10.1002/PAT.248.

[ref38] Vaganova E.; Wachtel E.; Goldberg A.; Yitzchaik S. White Light and Heat Sensitivity in a Pyridine-Based Polymer Blend. J. Phys. Chem. C 2012, 116, 25028–25033. 10.1021/jp3076063.

[ref39] Nečas D.; Klapetek P. Gwyddion: An Open-Source Software for SPM Data Analysis. Cent. Eur. J. Phys. 2011, 10, 181–188. 10.2478/S11534-011-0096-2.

[ref40] Lautenschlager P.; Allen P. B.; Cardona M. Temperature Dependence Af Band Gaps in Si and Ge 15 FEBRUARY 1985. Phys. Rev. B 1985, 31, 2163–2171. 10.1103/PhysRevB.31.2163. 9936023

[ref41] Zoltowski P. On the Electrical Capacitance of Interfaces Exhibiting Constant Phase Element Behaviour. J. Electroanal. Chem. 1998, 443, 149–154. 10.1016/S0022-0728(97)00490-7.

[ref42] Jorcin J. B.; Orazem M. E.; Pébère N.; Tribollet B. CPE Analysis by Local Electrochemical Impedance Spectroscopy. Electrochim. Acta 2006, 51, 1473–1479. 10.1016/J.ELECTACTA.2005.02.128.

[ref43] Bard A. J.; Faulkner L. R. Electrochemical Methods : Fundamentals and Applications; Wiley: New York, 1980.

[ref44] Lvovich V. F. Impedance Spectroscopy; John Wiley & Sons, Inc.: Hoboken, NJ, USA, 2012. 10.1002/9781118164075.

[ref45] Nolas G. S.; Sharp J.; Goldsmid H. J. *Thermoelectrics*; Springer Series in MATERIALS SCIENCE; Springer Berlin Heidelberg: Berlin, Heidelberg, 2001; Vol. 45. 10.1007/978-3-662-04569-5.

[ref46] Shaik S.; Danovich D.; Joy J.; Wang Z.; Stuyver T. Electric-Field Mediated Chemistry: Uncovering and Exploiting the Potential of (Oriented) Electric Fields to Exert Chemical Catalysis and Reaction Control. J. Am. Chem. Soc. 2020, 142, 12551–12562. 10.1021/jacs.0c05128. 32551571

