# Supplementary material for: Polymer Gel with Tunable Conductive Properties: A Material for Thermal Energy Harvesting
Source: ACS Omega. 2022 Dec 13;7(51):47747–54. doi: 10.1021/acsomega.2c05301 (PMC9798393; doi:10.1021/acsomega.2c05301)
Supplement: Supplementary file 1 — ao2c05301_si_001.pdf [file ao2c05301_si_001.pdf]

Supporting information

Polymer gel with tunable conductive properties: a material for thermal energy harvesting

Evgenia Vaganova[1], Dror Eliaz[1], Gregory Leitus,[2] Aleksei Solomonov[1], Faina Dubnikova[3], Yishay Feldman[2], Irit Rosenhek-Goldian[2], Sidney R. Cohen[2], and Ulyana Shimanovich*[1]

[1]Department of Molecular Chemistry and Materials Science, Weizmann Institute of Science, Rehovot 7610001, Israel; evgenia.vaganov@weizmann.ac.il (E.V.); dror.eliaz@weizmann.ac.il (D.E.); aleksei.solomonov@weizmann.ac.il (A.S.);
[2] Chemical Research Support Department, Weizmann Institute of Science, Rehovot 7610001, Israel; gregory.leitus@weizmann.ac.il, Isai.Feldman@weizmann.ac.il; <irit.goldian@weizmann.ac.il; Sidney.Cohen@weizmann.ac.il
[3] Chemistry Department, The Hebrew University of Jerusalem, Jerusalem, 91904, Israel; faina.dubnikov@mail.huji.ac.il
*[1]Correspondence: ulyana.shimanovich@weizmann.ac.il (U.S.).

Time dependence of the voltage across the external capacitor (20s heat)

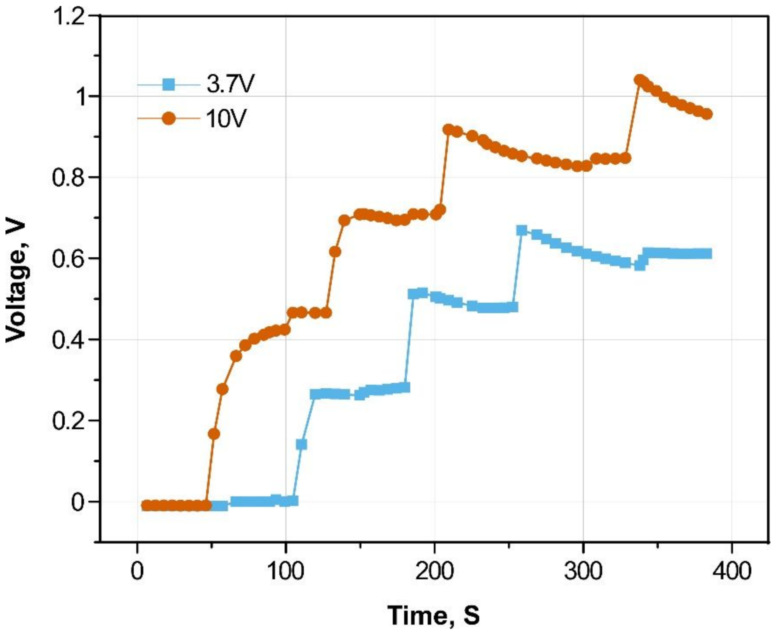

Figure S1. Time dependence of the voltage across the external capacitor, following 20s heating of the poly(4-vinyl pyridine)/pyridine gel sample: blue curve – 3.7V DC bias on the gel sample; red curve -10V DC bias on the gel sample.

Temperature dependence of the gel conductivity

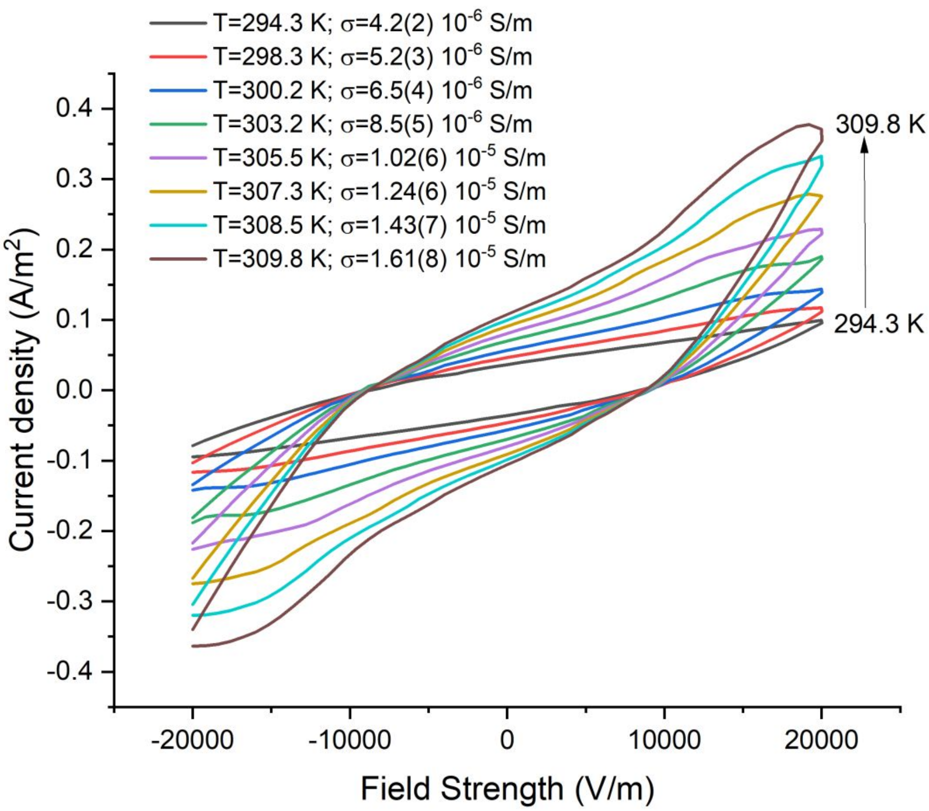

Figure S2. Conductivity changes of the P4VP gel under different temperature (range from 294 K till 310 K). Temperature was controlled by 336 Temperature Controller (LakeShore). (The voltage sweep rate 0.5V/s has been chosen).

Activation energy of the gel electrical conductivity

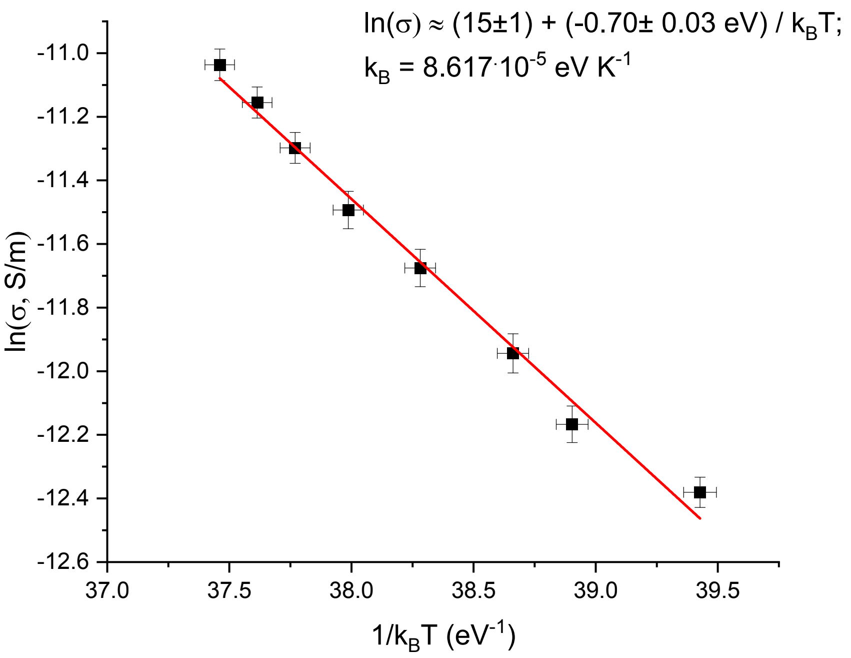

Figure S3. Arrhenius plot of the gel's conductivity. Activation energy of the electrical conductivity was found equal 0.7eV. This value in molar thermodynamic energy is ~64kJ/mol, which is consistent with the energy of *quasy- symmetrical* hydrogen bonded dimer ∼50kJ/mol.[1]

TEM images of the polymeric microspheres

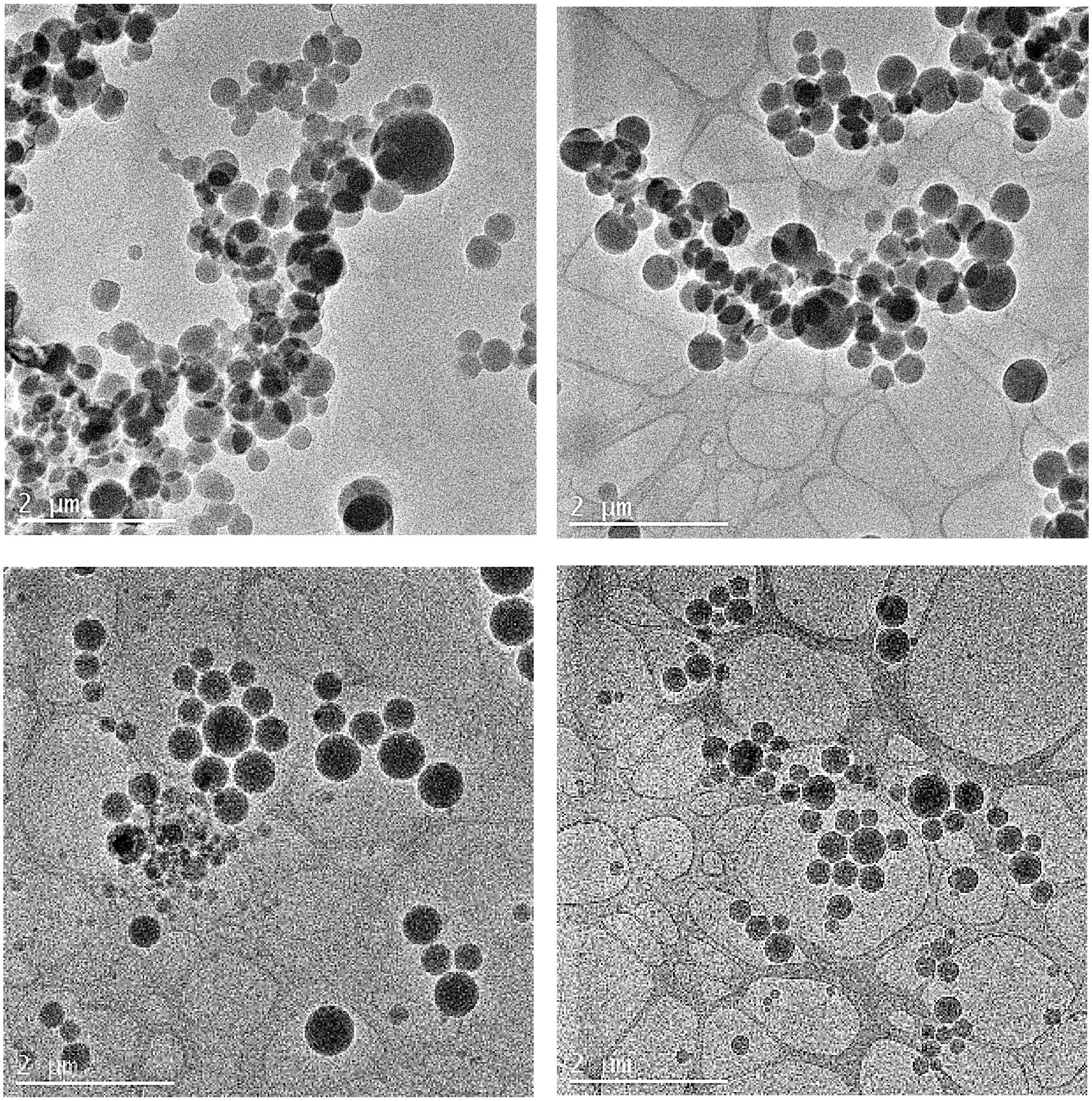

Figure S4. TEM images of the polymeric microspheres.

Images of height and phase of the gel microspheres.

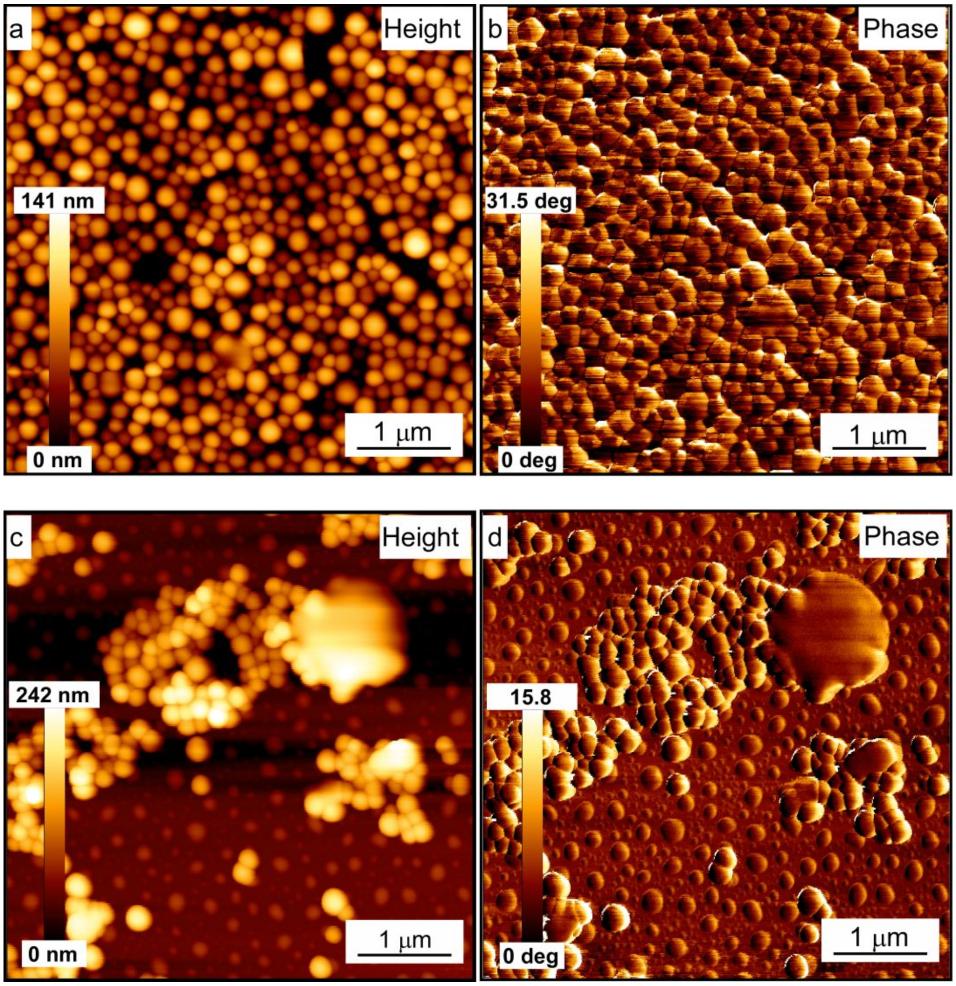

Figure S5. AFM images of PY4V gel. The height images (a and c) and the phase images (b and d). Scale bars are 1 μm for all images.

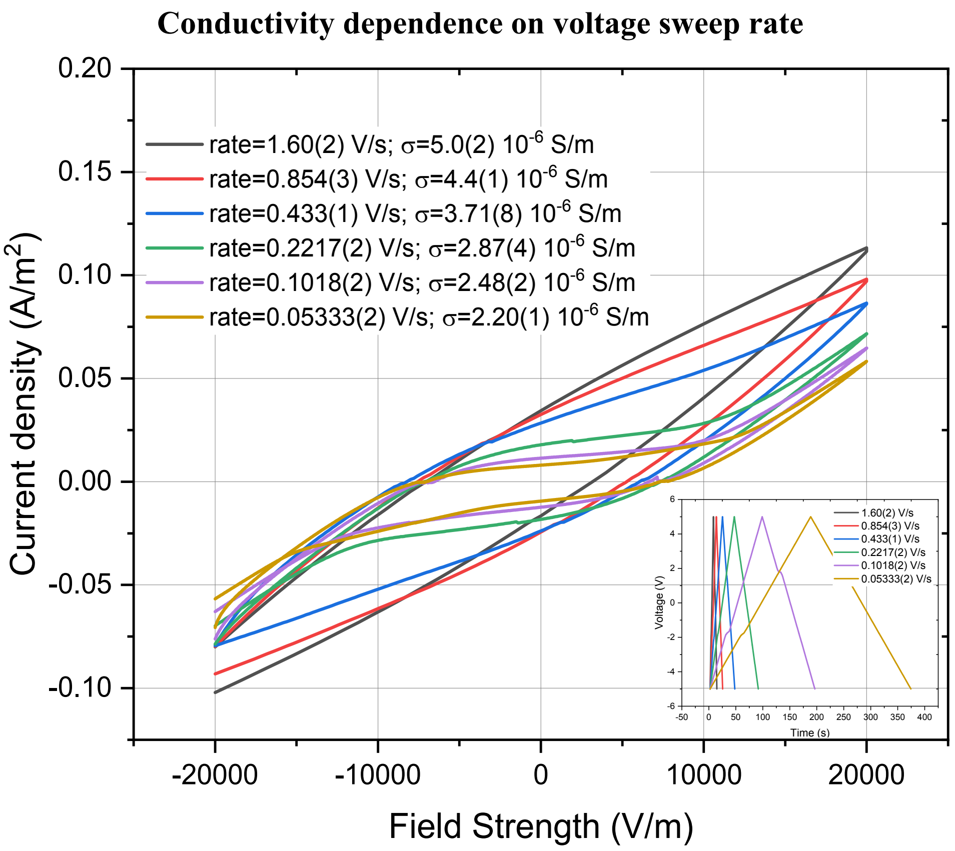

Figure S6. Changes in conductivity of the P4VP gel under different voltage sweep rate. Insert: time dependence of voltage on different linear voltage sweep rates.

FTIR spectrum of thin film of the P4VPy gel

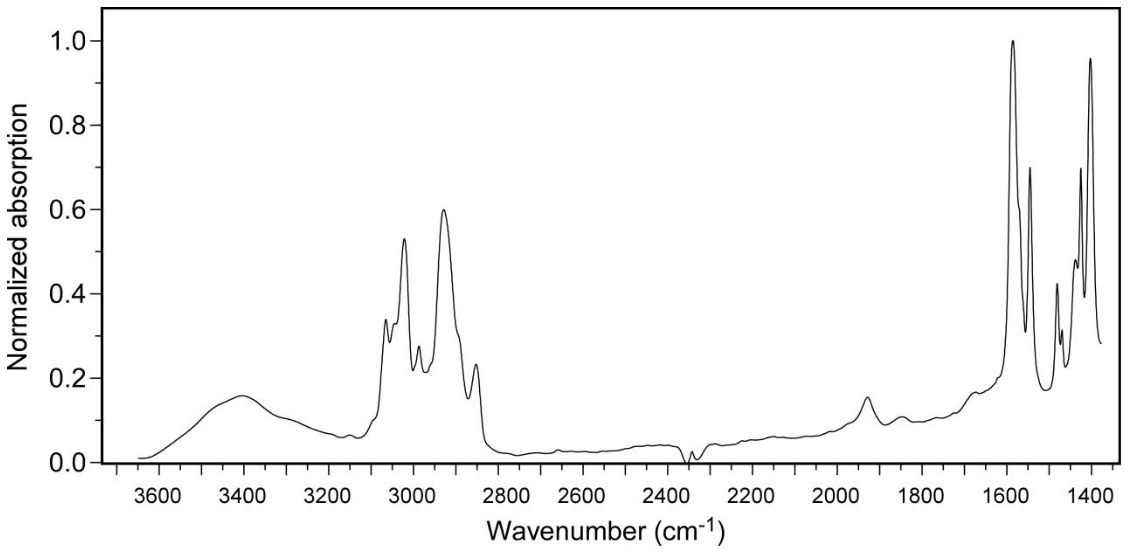

Figure S7. FTIR spectrum of the P4VPy gel. The experimental procedure of FTIR spectra measurements is described in [2] .

Mass spectroscopy spectra

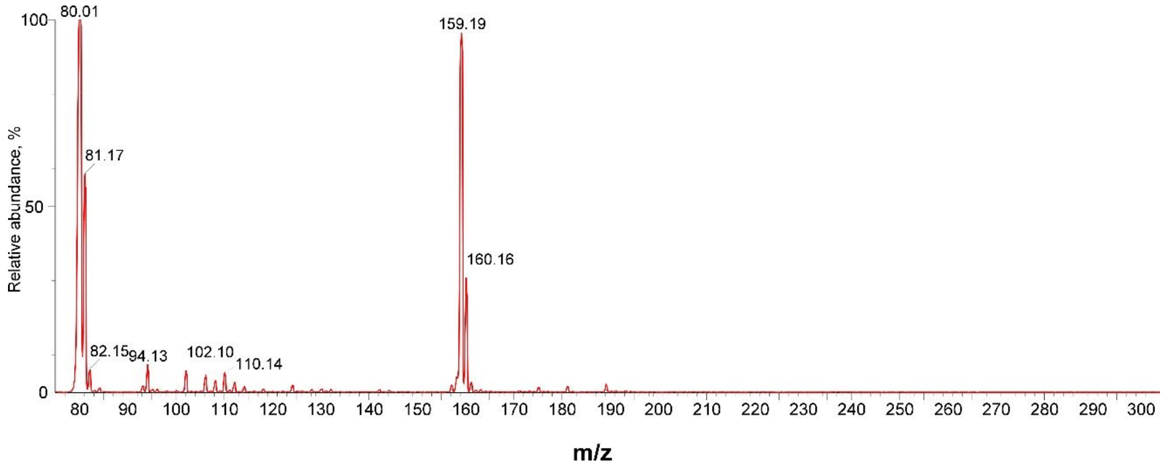

Figure S8. Mass spectroscopy spectra of 2-4 mg of a gel sample dissolved in 0.5ml MeOH in dark, m/z range is 75-300m/z. Mass spectroscopy samples and blanks (MeOH) were analyzed in both positive and negative ionization modes. For the negative ionization mode, no peaks appeared when the sample spectrum was compared to the blank spectrum, so the results are summarized only for the positive ionization mode. The peaks at 80.01m/z and 81.17m/z are assigned to pyridine and protonated pyridine molecules. The peaks at 159.19m/z and 160m/z indicate the presence of different dimers: $(Py)_2$ and (Py•••H•••Py),[1] respectively. The latter dimer is consistent with the formation of a quasi-symmetrical hydrogen-bonded dimer. Results were obtained at Cone voltage 20V.

DFT (Density Functional Theory) modeling (SI)

The optimized geometry structure and natural bond orbital (NBO) charge distribution of the P4VP monomer, the P4VP monomer with a protonated polymer side chain, and a neighboring liquid pyridine molecule were calculated using the B3LYP hybrid density functional in conjunction with the Pople 6-311++G(d,p) basis set, as implemented in the Gaussian-09 package.[3] An isopropyl pyridine (IPP4) was used as for a polymer side chain model.

Infra Red frequencies of the optimized protonated complex for comparison with experimental data [1,4] were calculated using the PBE1PBE hybrid density functional method [5] in conjugation with the Dunning correlation consistent polarized valence double ξ (cc-pVDZ) basis set[6].

Table S1. Summary of results of quantum mechanical calculations: ***(i)*** three single molecular structures: IPP4 – pyridine side chain; IPP4-sf – self-protonated pyridine side chain; pyridine. Dimers: a) protonated pyridine side chain ($RPyH^{+}$**)** hydrogen bonded (further bonded) with free pyridine (Py); and b) $RPyH^{+}$ bonded with side chain zwitterion (RC^{-}-PyH^{+}). ***(ii)*** N-H^{+} bonding energy for complex formation (kJ/mol) (minus sign represents an exothermic reaction), N-H^{+} stretching mode frequencies (cm^{-1}) and hydrogen bond distances between H and N atoms for two dimers (Å).

name	structure	bonding energy ΔE (kJ/mol)	$NH^{+}\cdots H$(Å)/N-H stretch (cm^{-1})
IPP4	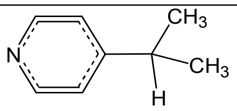		
IPP4-sp	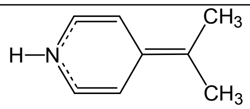		
pyridine	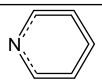		
Dimer A	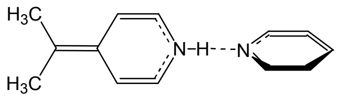	-28.5	1.98/3280
Dimer B	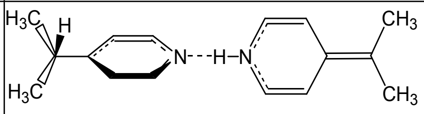	-30.1	1.98/3280

Molecules

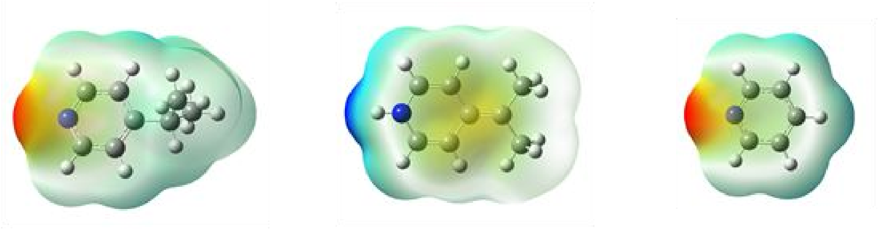

IPP4 IPP4-sp pyridine

(i) Dimers

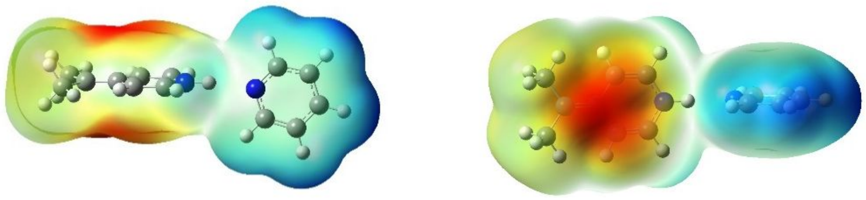

Dimer A (IPP4-sp …pyridine)

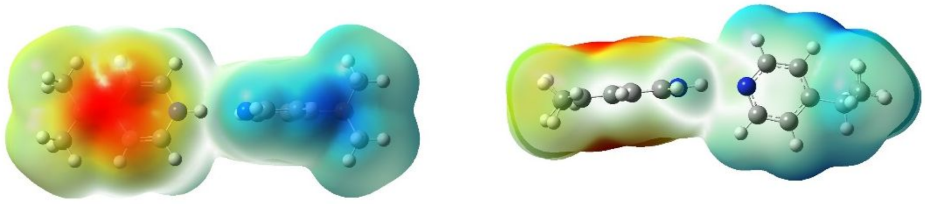

Dimer B (IPP4-sp … IPP4)

Figure S9. Charge distribution for free molecules and in two dimers. Blue denotes a positive and red denotes negative charges. sp stands for self-protonated.

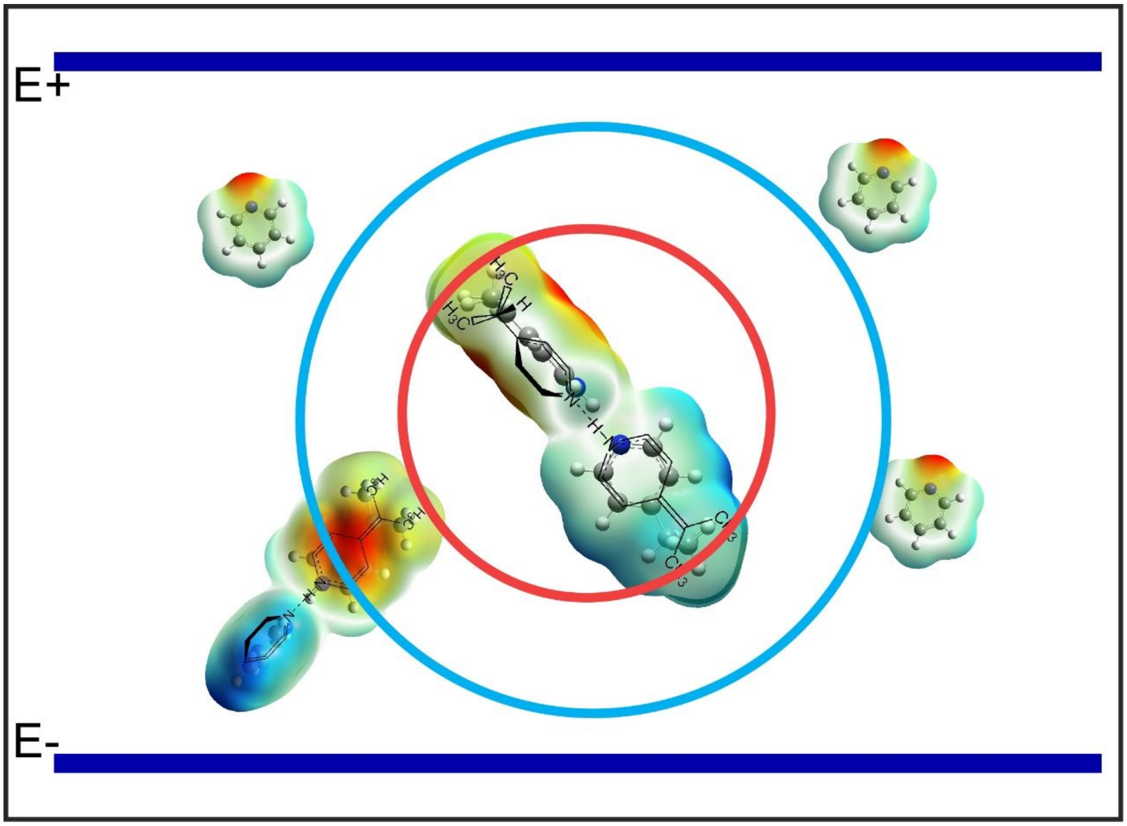

Figure S10. Illustrative sketch of the DC field-induced processes in microspheres. In the center of a microsphere, dimer IPP4-sp···IPP4, modelled PP4-sp/PP4, undergoes torque along the hydrogen bonding, aligning pyridine rings in a plane; IPP4-sp···pyridine, a model of PP4-sp/py, was found to be strongly polar[7] and ionized.

DFT (Density Functional Theory) modeling (SII)

Weakly hydrogen-bonded complex of a self-protonated pyridine side chain with pyridine dipole moments comparison in ground and excited states was studied to evaluate the DC field effect. The model of the complex (sketch below), was optimized by the B3LYP/cc-pVTZ level of theory with dispersion correction of GD3BJ. Ground state was calculated as singlet state S_0. The first triplet state (T_0) and first singlet state (S_1) were calculated as excited states. For calculations of the excited states (T_0 and S_1) we used the TDDFT[8] method as implemented in the Gaussian16 program.[3] Ionization potential was obtained using electron propagator theory (OVGF).[9] PP4-sp/py ionization potential, equal to

5.7eV, was much lower than for compare to pyridine (9.27eV) and the self-protonated pyridine side-chain (6.27 eV).[10]

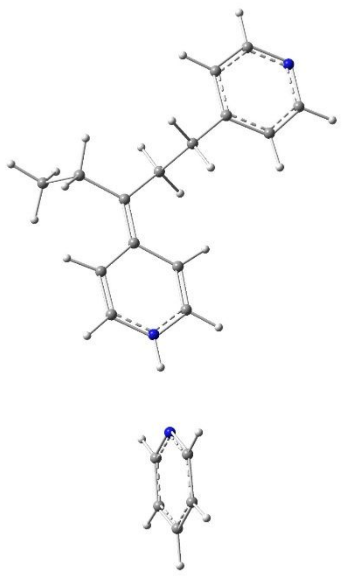

Results of our calculations presented below (**Table S2**). The difference between each state and S_0 shown in parentheses.

Table S2. Calculated energy of the states (eV) and Dipole moments (D)

State	Energy	Dipole moment
S_0 =	-940.6282471 au (0.0 eV)	10.1295 D
T_0 =	-940.554292 au (2.0124 eV)	20.3486 D
S_1 =	-940.554282 au (2.0127 eV)	20.3499 D

Boltzmann probability distribution

The Boltzmann distribution, yielding the probability φ_i of the system in a state *i* with energy E_i returns its statistical weight, i.e., concentration.

For systems with constant volume, temperature, and number of particles, the statistical weight of a state (φ_i) given by the Boltzmann factor follows [11]:

$$\varphi_i \sim e^{\left(\frac{-E_i}{RT}\right)}$$

where RT is 0.6 kcal/mol, E_i - energy of the weak H-bonds of the self-protonated side-chain/pyridine (PP4-sp/py) complex within the range ~ 4-8kJ/mol (0.96 – 1.91kcal/mol).

Therefore, the concentration of such complexes in the gel should be in the range of 4-20%.

REFERENCES

(1) Rozenberg, M.; Vaganova, E.; Yitzchaik, S. FTIR Study of Self-Protonation and Gel Formation in Pyridinic Solutions of Poly(4-Vinylpyridine). *New J. Chem.* **2000**, *24* (3), 109–111. https://doi.org/10.1039/A906937B.

(2) Nidhi Aggarwal, N.; Eliaz, D.; Cohen, H.; Rosenhek-Goldian, I.; Cohen, S.R.; Kozell, A.; Mason, T. O.; Shimanovich, U. *Communication Chemistry*, **2021**, *4*, (62), 1-10. | https://doi.org/10.1038/s42004-021-00494-2

(3) Frisch, M. J.; Trucks, G. W.; Schlegel, H. B.; Scuseria, G. E.; Robb, M. A.; Cheeseman, J. R.; Scalmani, G.; Barone, V.; Mennucci, B.; Petersson, G. A.; Nakatsuji, H.; Caricato, M.; Li, X.; Hratchian, H. P.; Izmaylov, A. F.; Bloino, J.; Zheng, G.; Sonnenberg, J. L.; Hada, M.; Ehara, M.; Toyota, K.; Fukuda, R.; Hasegawa, J.; Ishida, M.; Nakajima, T.; Honda, Y.; Kitao, O.; Nakai, H.; Vreven, T.; Montgomery, J. A.; Jr.; Peralta, J. E.; Ogliaro, F.; Bearpark, M.; Heyd, J. J.; Brothers, E.; Kudin, K. N.; Staroverov, V. N.; Kobayashi, R.; Normand, J.; Raghavachari, K.; Rendell, A.; Burant, J. C.; Iyengar, S. S.; Tomasi, J.; Cossi, M.; Rega, N.; Millam, J. M.; Klene, M.; Knox, J. E.; Cross, J. B.; Bakken, V.; Adamo, C.; Jaramillo, J.; Gomperts, R.; Stratmann, R. E.; Yazyev, O.; Austin, A. J.; Cammi, R.; Pomelli, C.; Ochterski, J. W.; Martin, R. L.; Morokuma, K.; Zakrzewski, V. G.; Voth, G. A.; Salvador, P.; Dannenberg, J. J.; Dapprich, S.; Daniels, A. D.; Farkas, Ö.; Foresman, J. B.; Ortiz, J. V.; Cioslowski, J.; Fox, D. J. Gaussian 16 Rev. B.01 https://gaussian.com/relnotes_b01/.

(4) Vaganova, E.; Rozenberg, M.; Yitzchaik, S. Multicolor Emission in Poly(4-Vinyl-Pyridine) Gel. *Chem. Mater.* **2000**, *12* (2), 261–263. https://doi.org/10.1021/cm990480x.

(5) Hartley, M. K.; Vine, S.; Walsh, E.; Avrantinis, S.; Daub, G. W.; Cave, R. J. Comparison of Relative Activation Energies Obtained by Density Functional Theory and the Random Phase Approximation for Several Claisen Rearrangements. *J. Phys. Chem. B* **2016**, *120* (8), 1486–1496. https://doi.org/10.1021/ACS.JPCB.5B06646/ASSET/IMAGES/LARGE/JP-2015-06646G_0003.JPEG.

(6) Dunning, T. H. Gaussian Basis Sets for Use in Correlated Molecular Calculations. I. The Atoms Boron through Neon and Hydrogen. *J. Chem. Phys.* **1998**, *90* (2), 1007. https://doi.org/10.1063/1.456153.

(7) Vaganova, E.; Wachtel, E.; Leitus, G.; Danovich, D.; Lesnichin, S.; Shenderovich, I. G.; Limbach, H.-H.; Yitzchaik, S. Photoinduced Proton Transfer in a Pyridine Based Polymer Gel. *J. Phys. Chem. B* **2010**, *114* (33), 10728–10733. https://doi.org/10.1021/jp104277r.

(8) Adamo, C.; Barone, V. Toward Reliable Density Functional Methods without Adjustable Parameters: The PBE0 Model. *J. Chem. Phys.* **1999**, *110* (13), 6158. https://doi.org/10.1063/1.478522.

(9) Zakrzewski, V. G.; Ortiz, J. V. Semidirect Algorithms in Electron Propagator Calculations. *Int. J. Quantum Chem.* **1994**, *52* (S28), 23–27. https://doi.org/10.1002/QUA.560520806.

(10) Vaganova, E.; Berestetsky, N.; Yitzchaik, S.; Goldberg, A. Modelling of Poly(4-Vinyl Pyridine) and Poly(4-Vinyl Pyridine)/Pyridine Composites: Structural and Optical Properties. *Mol. Simul.* **2008**, *34* (10–15), 981–987. https://doi.org/10.1080/08927020802256041.

(11) Rubinstein, Michael; Colby, R. H. *Polymer Physics*; Oxford, University Press, 2003.
